# Mitochondria at the Nanoscale: Physics Meets Biology—What Does It Mean for Medicine?

**DOI:** 10.3390/ijms25052835

**Published:** 2024-02-29

**Authors:** Lev Mourokh, Jonathan Friedman

**Affiliations:** Physics Department, Queens College, The City University of New York, 65-30 Kissena Blvd. Flushing, New York, NY 11367, USA

**Keywords:** mitochondria, respiratory transport chain, proton-pumping complex, equations of motion, carcinogenesis, autism spectrum disorder

## Abstract

Mitochondria are commonly perceived as “cellular power plants”. Intriguingly, power conversion is not their only function. In the first part of this paper, we review the role of mitochondria in the evolution of eukaryotic organisms and in the regulation of the human body, specifically focusing on cancer and autism in relation to mitochondrial dysfunction. In the second part, we overview our previous works, revealing the physical principles of operation for proton-pumping complexes in the inner mitochondrial membrane. Our proposed simple models reveal the physical mechanisms of energy exchange. They can be further expanded to answer open questions about mitochondrial functions and the medical treatment of diseases associated with mitochondrial disorders.

## 1. Introduction

A mitochondrion (plural: mitochondria) is an organelle with a size between 0.75 and 3 μm and is present in most eukaryotic cells. The mitochondrion is called the “powerhouse of the cell” [1] because it converts chemical energy from food into adenosine triphosphate (ATP), a molecular “energy currency” used in many cell functions throughout organisms. The energy conversion process in mitochondria starts in the cell matrix with the tricarboxylic acid cycle (TCA, also known as the Krebs cycle or the citric acid cycle), a series of chemical reactions that release stored energy through the oxidation of acetyl-coenzyme A molecules, which are derived from carbohydrates, fatty acids, and proteins. The TCA cycle reduces nicotinamide adenine dinucleotide (NAD^+^) to NADH, consumes acetate and water, and releases carbon dioxide. The NADH generated by the TCA cycle is supplied into the oxidative phosphorylation (OXPHOS) pathway, and it donates electrons to the mitochondrial electron transport chain. The machinery for OXPHOS is located in the inner mitochondrial membrane, where electron energy is used in proton-pumping complexes to create and maintain a proton population gradient. The resulting protonmotive force facilitates the mechanical rotation of the F_0_-F_1_ complex, producing ATP. The coupling of electron and proton transfer events to ATP production is at the center of the chemiosmotic theory proposed by Peter Mitchell [2], which earned him the Nobel Prize in 1978.

**Figure 1:**
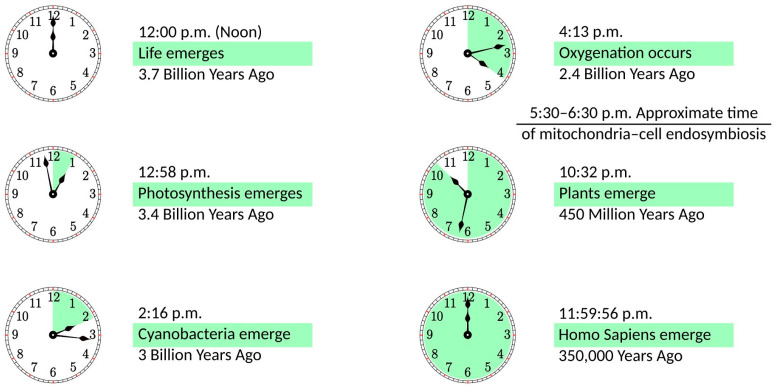
The history of life on a 12 h scale, with life emerging at noon and the present time being at midnight.

In the distant past, an ancestor of modern mitochondria was engulfed by the protocell and, rather than becoming digested, remained in a symbiotic relationship [3,4]. The importance of this event for the development of life on Earth can be illustrated in the 12 h scale shown in Figure 1. If life emerges at noon, it takes less than an hour to develop photosynthesis. Cyanobacteria appear at a quarter past two, and they need less than two hours to create an oxygen-rich atmosphere. Oxygenation is followed by a notable delay of more than six hours until plants emerge. Finally, with a vast acceleration of events, evolution goes from dinosaurs to mammals, primates, and *Homo Sapiens*, whose whole history takes just four seconds on this scale. It can be argued that this acceleration period is caused by mitochondria becoming fully operational, providing more energy for living organisms. In some sense, this development parallels the Industrial Revolution when the exponential growth of production was facilitated by coal mining, i.e., using a very condensed form of energy. Incidentally, mitochondria are fully functional without the host cell. They maintain their composition, organization, membrane potential, and ability to fuse [5]; they are fully competent for respiration and ATP synthesis [6], as well as for protein import [7]. The same cannot be said about the host organism, as it would not survive without mitochondria.

In addition to energy conversion, mitochondria play many other roles in cell operations. They act in calcium signaling [8], stress response [9], and stem cell regulation [10,11] and also serve as general cellular signaling hubs [12]. They regulate aging [13] and control cell death (apoptosis) [14]. Given these functions, human health strongly depends on proper mitochondrial operation. In addition to specific mitochondrial disorders [15], there are links between mitochondrial dysfunction and many pathologies, including Parkinson’s, Alzheimer’s, and Huntington’s diseases [16]. Mitochondrial dysfunction is known to account for the development of most cardiovascular illnesses [17]. Even the resistance to SARS-CoV-2 is shown to be related to mitochondrial health [18].

In this review, we discuss two specific topics related to mitochondrial disorders. The role of mitochondria in carcinogenesis is addressed in Section 2, while in Section 3, we overview the possible relationship between mitochondria and the origin of autism. We have chosen carcinogenesis and autism for two reasons. First, they have been previously discussed but still need to be appreciated further, and second, our prior studies (reviewed in Section 4) can be readily extended to examine them. In Section 5, we present the conclusions of our work.

## 2. Mitochondria and Carcinogenesis

### 2.1. General Discussion

Cancer is a major public health problem worldwide and the second leading cause of death in the United States, with an estimated 609,360 people dying from cancer in 2022, corresponding to almost 1700 deaths per day [19]. Billions of dollars have been spent on cancer-related research and trillions more on treatment. Still, despite certain improvements in cancer survival rates and the development of successful treatment methods for certain types of cancer, the War on Cancer is still far from victory. Diagnostics and treatments are primarily aimed at the latter stages when carcinogenesis becomes almost irreversible. To shift the paradigm, efforts should focus on precancerous changes, developing approaches to determine these changes and return to a healthy state.

Currently, the dominant concept of carcinogenesis is somatic mutation theory [20]. It states that a single “renegade cell” [21] acquires a set of sufficiently advantageous mutations that allows it to immortalize, proliferate autonomously, invade tissues, and metastasize. However, carcinogens cannot affect the cell’s nucleus directly, except for high-frequency irradiation. The immune system provides the initial response to carcinogens, and because of “overhealing” [22,23], the extracellular matrix (ECM) is altered, affecting bioelectric signaling [24]. The normal wound healing includes ECM remodeling and the formation of new tissues (scars). The overproduction of the cells and other factors involved in these processes, especially macrophages [23], can lead to overhealing and the start of carcinogenesis. The idea that ECM alteration is the origin of carcinogenesis was previously suggested [25,26], but the pathway from this starting point to DNA mutations was either ignored [25] or vaguely described [26]. We believe that mitochondria play a crucial role as a mediator between the ECM and the cell nucleus, and monitoring and controlling this relationship can be important for cancer prevention and treatment. Mitochondrial defects can include aberrated metabolism (the Warburg effect) or signaling dysfunction (reactive oxygen species or ROS production). We discuss these two mechanisms below.

### 2.2. Warburg Effect

Historically, the Warburg effect was mitochondria’s first and solid connection to carcinogenesis. In the presence of oxygen, mitochondria perform oxidative phosphorylation as the primary metabolic pathway. Alternatively, when oxygen is limited, anaerobic fermentation can metabolize glycolytic products, with lactate as a final product. The second pathway is much less effective, producing only two ATP molecules per glucose molecule, while OXPHOS can make 30 to 36 ATP molecules [27]. Over one hundred years ago, Otto Warburg observed that cancer cells use fermentation as the primary metabolic pathway, even in the presence of oxygen, and this process is called aerobic glycolysis [28,29]. He suggested that the associated mitochondrial dysfunction is the cause of cancer [30]. However, this hypothesis has been challenged in recent years due to findings that upregulated glycolysis in many cancers is not accompanied by detectable mitochondrial defects or OXPHOS disruptions [31,32]. In addition, there is evidence that the upregulation of glycolysis is not just for ATP synthesis but also for producing biomasses such as ribonucleotides [33] and amino acids [34].

With the general conclusion that cancer does not inactivate mitochondrial functions but instead alters its bioenergetic and biosynthetic state [35], the Warburg effect was studied concerning its role in cell signaling [36,37]. In particular, researchers uncovered its role in ROS regulation and redox balance [38,39], histone acetylation level [40,41], and oncogene-induced senescence [42]. However, in all cases, the Warburg effect is a consequence of tumorigenesis rather than its origin, as cancer cells actively reprogram their microenvironment.

### 2.3. ROS Production

The chemical reduction of O_2_ forms reactive oxygen species (ROS), including superoxide (O_2_^−^), hydrogen peroxide (H_2_O_2_), and the hydroxide (OH^−^). For many years, the perception of ROS was solely destructive, as they were associated with oxidative stress and thought to induce pathology generally by damaging lipids, proteins, and DNA [43]. Quite recently, it has become evident that ROS contribute to intracellular signaling to control numerous physiological and pathological cell processes [44,45]. The interplay of the damaging effects and regulatory functions of ROS has been discussed in several reviews [46,47,48].

The primary source of ROS within a cell is mitochondria [49]. The electron transport chain is a producer of ROS, with the electron leakages from Complexes I, II, and III creating O_2_^−^ by single-electron oxygen reduction [50,51]. Under physiological conditions, it is estimated that 0.2 to 2% of leaked electrons do not contribute to ATP production [52]. While Complexes I and II exclusively create O_2_^−^ in the mitochondrial matrix, Complex III produces O_2_^−^ in both the matrix and intermembrane space [53,54]. In the latter case, O_2_^−^ travels through voltage-dependent channels in the outer mitochondrial membrane and into the cytosol [55], where it can be converted into H_2_O_2_, participating in cellular signaling events [56].

Oxidative DNA damage can be a significant contributor to cancer [57]. In particular, the reaction of OH^−^ with DNA accounts for most DNA strand breaks, representing the primary molecular reaction leading to carcinogenesis. To defend from the damage, cells use various antioxidant mechanisms, such as converting highly reactive O_2_^−^ into H_2_O_2_ using superoxide dismutases [58]. However, a high concentration of H_2_O_2_ is also detrimental since H_2_O_2_ can be reduced to the damaging hydroxides OH^−^ in the presence of Fe^2+^ and Cu^2+^ [59]. Producing the amount of ROS needed for self-signaling without harmful overproduction requires a delicate balance of various components, and its imbalance can mediate damage transfer from the ECM to DNA. One of the pathways to ROS overproduction can be aberrated bioelectric signaling in the ECM, increasing the mitochondrial membrane potential [60].

The involvement of mitochondria in the process of carcinogenesis is evident. However, the details are presently unclear, and we believe that understanding the physical mechanisms of operation for various parts of mitochondrial machinery is necessary. In particular, our models of the mitochondrial proton-pumping complexes described in Section 4 can be extended to consider electron leakage, corresponding ROS production, and conditions for their possible overproduction. This would be an essential step toward understanding carcinogenesis, potentially leading to a paradigm shift in early diagnosis and treatment.

## 3. Mitochondria and Autism: Quantum Brain Hypothesis

### 3.1. Link between Autism and Mitochondrial Disorder

People with autism spectrum disorder (ASD) are characterized by impaired social interaction and communication, as well as a deficiency in learning ability. It is a life-long condition, with symptoms generally appearing in early childhood. There is currently no cure for ASD, and the underlying reasons remain unclear. The most promising hypotheses include genetic predisposition, epigenetic modifications, nutritional influences, and exposure to environmental toxins during critical development periods [61,62].

Growing evidence implies that ASD is linked to mitochondrial dysfunction [63,64,65,66]. The earliest hint appears in [67], where an elevated level of lactate was observed in the plasma of autistic patients, indicating a defect in oxidative phosphorylation. The concept of ASD as a mitochondrial disorder was proposed in [68]. It was based on indirect evidence such as lactic acidosis, elevated urine levels of Krebs cycle metabolites, plasma carnitine deficiency, decreased brain glucose utilization, and decreased ATP levels in autistic patients. The perception of bioenergetic deficiency in children with ASD was further supported by detecting various abnormal biomarkers in the brain, plasma, cerebral spinal fluid, urine, fibroblasts, skeletal muscle, and buccal mucosa [66,69]. There was also direct evidence of the impairment of proton-pumping complexes of the electron transport chain (especially Complex I) in mitochondria from skeletal muscle [70,71] and the brain [72,73] for autistic patients. Furthermore, the condition called “mitochondrial hyperproliferation”, where children have high numbers of mitochondria but reduced energy output from these organelles, is connected to ASD [74].

The links discussed above are related to the bioenergetic functions of mitochondria. However, ASD can be affected by other mitochondrial functions, such as oxidative stress via ROS production or defects in calcium homeostasis. In particular, evidence of increased oxidative damage to DNA, proteins, and lipids has been identified in blood, urine, and postmortem brain samples from autistic individuals [75]. In this review, we propose another possible link between mitochondrial dysfunction and ASD using the quantum brain hypothesis.

### 3.2. Quantum Cognition

The idea that quantum correlations play a role in the brain’s functioning was first proposed by Roger Penrose [76]. It was swiftly dismissed because of the common belief that such correlations cannot survive in the “hot and wet” brain environment [77]. Recently, the notion of a quantum brain was revived in [78] by the suggestion that quantum correlations can be stored in nuclear spins with extremely long decoherence times (in contrast to previously proposed microtubules [79]). The idea is that when the adenosine triphosphate (ATP) molecule is dissociated into adenosine monophosphate (AMP) and a pyrophosphate ion (P_2_O_7_^4−^), the two phosphorous nuclei are in the singlet state, forming biological qubits, as the nuclei are in the entangled spinup–spindown superposition. These nuclei remain entangled when the pyrophosphate ion is subsequently dissociated into two phosphate ions (PO_4_^3−^). Finally, these two phosphate ions separately join with calcium ions to form two Posner molecules (Ca_9_(PO_4_)_6_), making them entangled. Posner molecules appear in two different presynaptic neurons and melt simultaneously because of the entanglement. This leads to the simultaneous injection of Ca^2+^ ions and activation of the neurotransmitters in the two presynaptic neurons, introducing correlations that are assumed to be transferred to the postsynaptic neurons and their correlated firings. In other words, it was suggested that a quantum correlation between phosphorous nuclear spins might lead to a correlation in operation between different neurons. Later, it was shown [80] that such correlations enable full-scale quantum computations in the brain. Although it was recently demonstrated [81] that the Posner molecule, as a trimer, is not stable enough to host the biological qubit, the corresponding dimer can serve instead [81].

Mitochondria, which both produce ATP and host calcium stores, can be sites for the formation of Posner molecules or associated dimers. To produce the pyrophosphate molecule necessary for phosphorous spin entanglement, two phosphate groups should be detached during ATP hydrolysis instead of a single one in the usual ATP-ADP cycle. This double phosphate group detachment occurs in the synthesis of cAMP (cyclic AMP) from ATP by adenylyl (adenylate) cyclase. cAMP is an important secondary messenger crucial for intracellular signaling [82] and has been studied for decades. In particular, it was shown [83,84] that cAMP is generated inside mitochondria by the action of soluble adenylyl cyclase, and this reaction produces the pyrophosphates that drive quantum brain computation.

### 3.3. Quantum Cognition and Autism

The following set of unproven statements are worth pursuing as directions for further research and might usher in a basis for the early determination of pre-ASD conditions. Operations of the human brain are not purely classical or purely quantum but rather a combination of both. Each specific brain function can require either quantum or classical cascades. A child’s brain learns to apply necessary tools for certain operations in the first several years of human life. The proper balance of quantum and classical cognition is essential throughout early childhood. When this balance is broken, aberrations in brain development will arise. In particular, when quantum processes prevail, ASD forms. Persons with ASD can be very advanced in fields requiring quantum computations, as they can manipulate huge numbers or remember an enormous amount of music. However, when classical computations are necessary, as in learning basic skills or communications, the situation becomes challenging. Improper functioning of mitochondria (the wrong ratio of produced phosphates and pyrophosphates) can be a reason for the unbalanced inclination toward quantum computations in the brain. If they are detected and treated, the preconditions of ASD can be eliminated.

Our hypothesis that mitochondria provide the molecular basis for quantum cognition is clearly more speculative than the role of mitochondria in carcinogenesis discussed in the previous section. Moreover, the connection between the prevalence of quantum computations in the human brain and ASD is entirely unproven. However, we believe that studies in this direction can be vital. They will lead to a better understanding of brain functions, mechanisms for their aberrations, even the origin of cognition, and possible prevention and treatment of ASD. In this, revealing the physical principles of the interaction between mitochondria and neuronal networks beyond energy production is crucial. Simple physical models of the various mitochondrial operations described in Section 4 can be readily extended to incorporate the ATP-cAMP reaction as a starting point for quantum correlations in the brain. Determination of the dynamics of this process and, especially, the production rate of the entangled phosphorous nuclei will shed light on the feasibility of the quantum computations in the brain. Moreover, it can find the conditions for a proper balance between classical and quantum cascades in neuronal networks, i.e., help to diagnose, treat, or even prevent ASD if our hypothesis is validated.

## 4. Proton-Pumping Complexes of the Inner Mitochondrial Membrane: Physical Principles of Operation

### 4.1. General Approach

**Figure 2:**
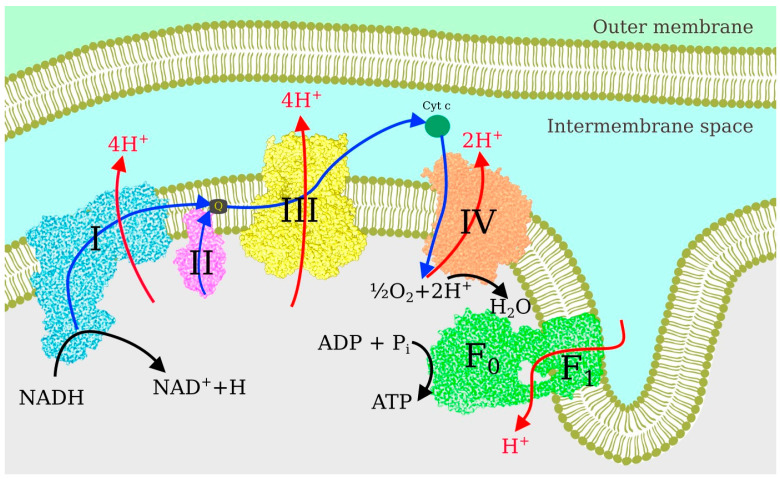
Electron transport chain in the inner mitochondrial membrane.

Following the TCA cycle in the mitochondrial matrix, the respiratory chain of the inner mitochondrial membrane converts and stores energy in ATP molecules via the OXPHOS process [85]. The sequence of events is illustrated in Figure 2. Each electron donated by NADH has more than one electron volt of excess energy, which can be easily lost to heat if not converted into a more stable form. In the first step, performed by the proton-pumping Complexes I, III, and IV, the electron energy is used to move protons from the negative side of the membrane with a small proton population to the positive side with a large proton population, i.e., to pump them against the chemical potential. In the second step, the resulting proton current through the F_0_–F_1_ complex facilitates the rotation of the nanomotor, i.e., the energy is converted into mechanical form. In the third step, mechanical energy combines ADP and an inorganic phosphate molecule to form ATP.

Electron transport along the chain occurs in several forms. Inside the protein complexes, electrons jump from one metal atom (or FeS complex) to another. From Complex I to Complex III, electrons ride quinol shuttles, which move inside the lipid membrane. The polarized lipid membrane significantly suppresses the diffusion of charged objects. To preserve charge neutrality, quinols are populated by electrons and protons, which actuates proton pumping, as discussed in Section 4.4 below. Electrons are transferred from Complex III to Complex IV using cytochrome c as a shuttle. Cytochrome c moves outside the membrane and, accordingly, can be in reduced form.

The single-particle nature of electron transport has pronounced similarities to semiconductor nanodevice operation, including similar energy scales. The total energy drop in the mitochondrial electron transport chain is about 1.1 eV (in several steps), while quantum transport in semiconductor nanostructures can be seen at cryogenic temperatures with energy level separation typically in the meV range. Correspondingly, the energy scales with temperature. Moreover, as the electron transfer event facilitates proton pumping, the energy exchange can be described as a two-particle Coulomb interaction. We believe that condensed matter and statistical physics approaches, which are well-established for artificial nanostructures, can be applied to living objects at the nanoscale. If we consider quantum dots as artificial atoms, we can think of atoms as natural quantum dots.

In the following sections, we discuss simple models for each proton-pumping complex of the inner mitochondrial membrane to reveal the physical mechanisms of operation. For our calculations, we start with the Heisenberg equation of motion for the electron and proton operators in the presence of the environment. Averaging over the environment and using a high-temperature approximation, valid for living objects, these equations can be rewritten as rate equations for electron and proton populations. Electron and proton currents can then be expressed in terms of these populations. Where a mechanical motion is involved (see Section 4.3 and Section 4.4), these rate equations are coupled to the empirical Langevin equation. Solving the coupled equations numerically, we can determine the proton and electron currents, their ratio (quantum yield), the efficiency of the energy transfer, and the dependencies of these quantities on the parameters of the system. The order of sections below is based on the complexity of calculations, from the simplest model to the most complicated one.

### 4.2. Complex IV, Cytochrome c Oxidase

**Figure 3:**
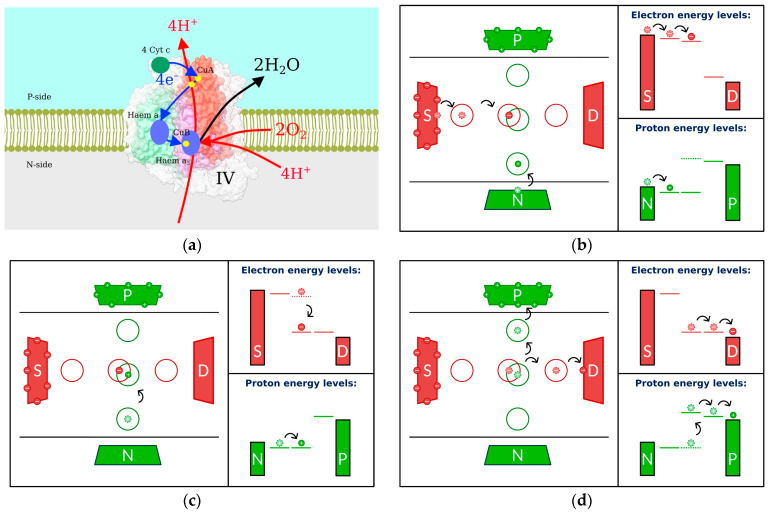
Complex IV: (**a**) structure; (**b**–**d**) sequence of events leading to proton pumping. Left: Transfer events. Right: Energetic schematics. Electrons are shown as small red solid circles with negative signs, and S and D denote electron source and drain reservoirs, respectively. Protons are shown as small green solid circles with positive signs, with N and P serving as reservoirs at the negative and positive sides of the membrane, respectively. Larger red and green circles represent the electron and proton sites, respectively. Electron and proton transfer events are shown by black arrows, with the former positions for electrons and protons appearing as dashed circles with lighter colors. Electron and proton energy levels are represented by solid horizontal lines. The former positions are dashed.

Electrons are brought to Complex IV (Figure 3a) by the cytochrome c shuttle and subsequently jump to the Cu atom, followed by the haem and the haem/Cu binuclear center [86]. After this last jump, an electron combines with a proton from the negative side of the membrane and with oxygen to form water. Simultaneously, an additional proton is transferred to the positive side of the membrane. In a series of papers [87,88,89,90], we proposed several models revealing the physical principles of operation for Complex IV. The most elaborate model [89] is shown in Figure 3b–d.

Electrons (shown as small red circles) move along the membrane from the source (S) to the drain (D) through three intermediate sites. Protons move across the membrane through three separate sites from negative (N) to positive (P) sides. The structure of the energy levels is such that the electrons are moving from higher to lower potential, and our goal is to use released energy to pump protons from lower to higher potential (tops of the reservoir-representing rectangles). The energy levels of the left electron site and the lower proton site are below the chemical potentials of the corresponding reservoirs, and initially, they are populated (Figure 3b). The electron can proceed to the middle site, but then it is stuck there temporarily because the energy separation between this level and the next one (to the right) is too large, and it is not easy to unload this energy into heat. Now that the middle electron site is populated, the energy of the middle proton site decreases due to the electron–proton electrostatic interaction, and a proton can progress to the middle site (Figure 3c). With the middle proton site populated, the energy of the middle electron site decreases, and the electron can escape to the next site and reservoir on the right. With the electron gone, the energy of the proton middle site increases (Figure 3d), allowing the proton to proceed to the upper site and, finally, the positive side of the membrane.

Using the approach described in the previous section, we determined the proton current, and our results show that proton pumping against the population gradient is possible under physiological conditions. Moreover, cell parameters have been tuned through evolution so that physiological temperatures are optimal for proton pumping. We compared the kinetic phases predicted by our simple model [90] to those measured in the experiment of [91] and found excellent agreement.

### 4.3. Complex I, NADH-Quinone Oxidoreductase

**Figure 4:**
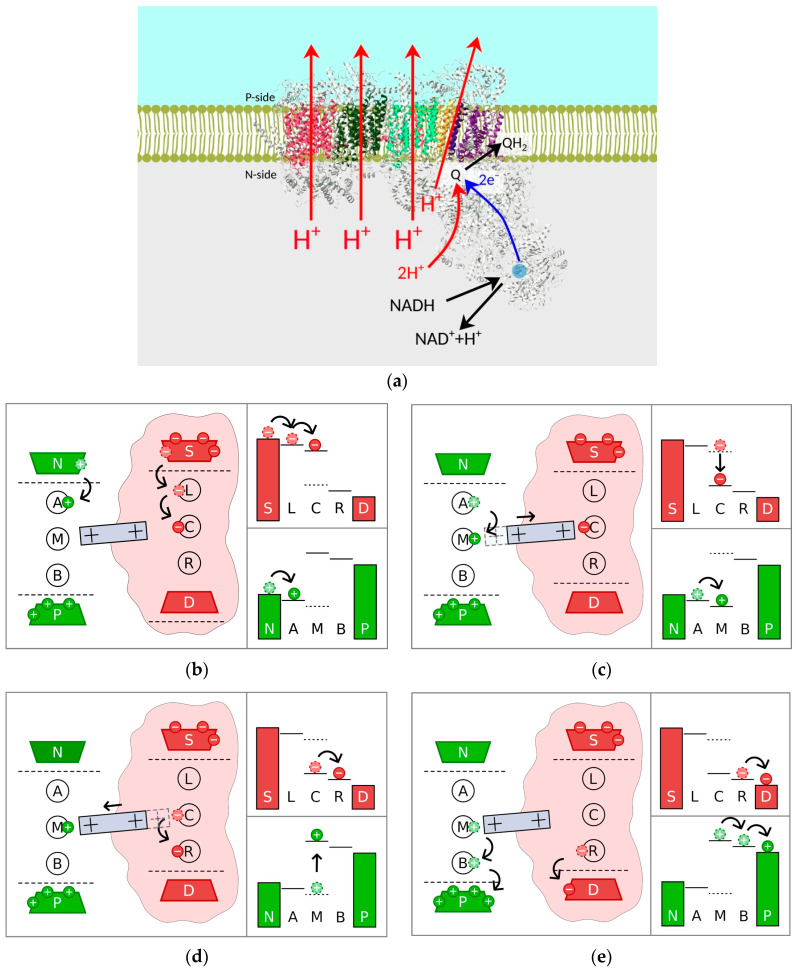
Complex I: (**a**) structure; (**b**–**e**) sequence of events leading to proton pumping. Shapes and schematics are the same as in Figure 3. L, C, and R are the electron sites, and A, B, and M are the proton sites. The grey rectangle with positive signs represents the piston, with the dashed part showing the former position.

Complex I has an L-shape, as shown in Figure 4a, with a hydrophobic arm embedded in the membrane and a hydrophilic arm extending into the matrix [92]. This is the beginning of the electron transport chain. The electrons donated by nicotinamide adenine dinucleotide (NADH) travel to quinone through the hydrophilic arm via a series of FeS complexes. In the model of [93], four proton pumps located at different subunits of the hydrophobic arm transfer protons across the membrane. In vast contrast to Complex IV, the electron and proton pathways here are well separated by a distance of tens of nanometers, prohibiting direct Coulomb interaction. However, it is commonly accepted that electron transfer creates a so-called electrostatic wave along the membrane arm, facilitating proton pumping [94]. Several physical mechanisms were suggested to account for this electrostatic wave. Initially, the mechanical motion of the lateral helix was proposed [94,95]. In the oxidized state, this helix moves to the right to open the upper half-channels for protons. In the reduced state, it moves to the left to open the lower half-channels. However, the role of the helix in proton pumping was not confirmed in mutation experiments [96]. An electrostatic wave mechanism based on molecular dynamics involves coordinated forward and backward waves of conformational changes and charge exchange [97]. Very recently [98], it was proposed that the rotation of the transmembrane helix near the quinone-binding site achieves the coupling of electron transport and proton pumps. It should be noted that in this work, it was also suggested that all the proton pumping occurs in the left-most subunit of the membrane arm, shown in red in Figure 4a.

In our work, we model the electrostatic wave as the motion of a piston, with positive charges at the edges and negative charges in the middle, as shown as a grey rectangle in Figure 4b–e [99]. It does not have a specific structural association and may represent the action of the transmembrane helix of [98]. We use a similar set of electron and proton sites and a similar (albeit different) structure of the energy levels, as compared to the model of Complex IV described above. Similar to that complex, electrons can move to the middle site (Figure 4b). According to our model, the electron attracts the positive charge from the right edge of the piston, and the piston moves to the right (Figure 4c). Consequentially, the positive charge at the left edge would move away from the middle proton site, decreasing its energy and allowing the proton to move there. At the same time, the positive charge at the right edge moves closer to the middle electron site, decreasing its energy and allowing the electron to escape into the reservoir (Figure 4d). When the electron is gone, elastic forces return the piston to the left, increasing the energy of the already populated proton middle site and facilitating proton pumping (Figure 4e).

In our calculations, the derived rate equations for the electron and proton populations are coupled to the Langevin equation describing the motion of the piston [99]. In particular, the energies of the middle sites depend on the piston position, while the electrostatic forces on the piston (and hence the piston position) depend on the middle sites’ populations. These coupled equations were solved numerically, and the conditions for proton pumping were determined. As in the case of Complex IV, optimal pumping is achieved at physiological temperatures.

### 4.4. Complex III, Coenzyme Q—Cytochrome c Reductase

**Figure 5:**
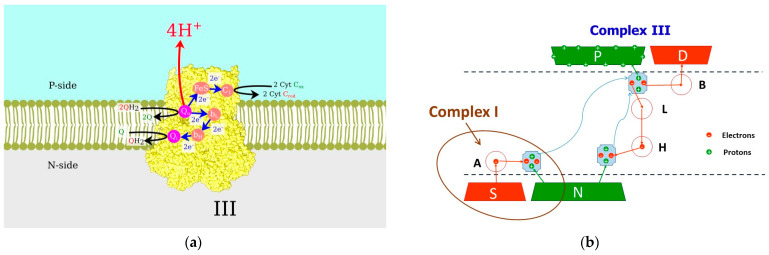
Complex III: (**a**) structure and Q-cycle operations; (**b**) proposed model of Q-cycle. Light blue shapes are the quinols. Electron and proton transfer events are represented by the red and green arrows, respectively. Blue curvy arrows show the diffusion paths of the quinols. Dashed lines represent the membrane boundaries.

While Complexes I and IV are purely mitochondrial, Complex III belongs to the family of the bc-complexes, which also includes bacterial cytochrome bc_1_ and cytochrome b_6_f in chloroplasts [100], where the so-called Q-cycle occurs [101] (shown in Figure 5a). Each quinone (Q) molecule is populated by two electrons and two protons at the N-side of the membrane in Complex I, undergoing reduction to quinol (QH_2_) molecules. Shuttles diffuse across the intramembrane space, and when they reach the Q_0_-site of Complex III, the protons are translocated to the P-side of the membrane, while the electrons follow different pathways. The first electron is accepted by the low-energy, iron–sulfur Rieske protein. Afterward, it proceeds to the mobile shuttle cytochrome c toward Complex IV, while the second electron is transferred via hemes b_L_ and b_H_ to the Q_i_-site (near the N-side). After the second turnover, two electrons and two protons populate the quinone from the Q_i_-site, and the resulting QH_2_ shuttle diffuses to the Q_0_-site.

Our model [102] is presented in Figure 5b. The electron pathway originates in reservoir S and terminates in reservoir D. Protons are transferred from reservoir N to P. Electron transport is downhill from the reservoir of higher energy (S) to the reservoir of lower energy (D). In contrast, the proton transfer is uphill against the electrochemical gradient across the membrane. The first quinone shuttle is populated by two electrons from site A, representing the quinone-binding site of Complex I. When the first electron is transferred to the shuttle, site A is immediately repopulated from reservoir S. With one electron on quinone, the energy of the proton level on the shuttle decreases, and it can be populated from the N-reservoir. After the process is repeated, the resulting quinol molecule diffuses across the membrane. When the first electron is unloaded to site B, which represents the FeS Rieske protein, the energy of the proton level on the shuttle increases, and it can proceed to the P-reservoir, with the same process for the second electron and proton pair. Two electrons moving through the L and H sites populate the second quinone and attract two more protons from the N-reservoir. The second quinol also diffuses across the membrane and unloads two electrons and two protons.

In our calculations [102], we derived the equations of motion for the electron and proton creation/annihilation operators at all sites and shuttles. Averaging these equations over the environment, we obtained rate equations for several coupled density matrices representing different system parts. These rate equations are coupled to phenomenological Langevin equations describing the motions of the shuttles, as electron and proton population/depopulation depends on the distance between the shuttle and the site or reservoir. We numerically solved the resulting set of 40 coupled equations and obtained the time-dependent positions of the two shuttles and the time-dependent electron and proton populations. We also calculated the number of translocated electrons and protons to determine the quantum yield and thermodynamic efficiency. We obtained a quantum yield of almost two for a short initial time, as can be expected from the ideal Q-cycle performance. With elapsed time and the two shuttles unloaded to the same sites without synchronization, the quantum yield value decreases but still significantly exceeds unity. The oversimplification of our model can explain the deviation from the expected quantum yield of two, as we only used two shuttles moving back and forth instead of a pool of quinones available in the natural system.

## 5. Conclusions

For years, biology and physics were entirely separate fields of science. Biology studies living organisms, including their origin, evolution, function, and structure. Physics studies matter, energy, their interaction, and dynamics. Biophysics initially emerged as studies of biological objects using methods of experimental physics. The first example can be the works of Luigi Galvany, and an illuminating illustration is the discovery of the double-helix DNA structure by Francis Crick and James Watson based on the X-ray diffraction patterns obtained by Rosalind Franklin and Maurice Wilkins. Other applications of the physics-based approaches, such as X-ray diffraction, nuclear magnetic resonance spectroscopy, atomic force microscopy, electron microscopy, and optical tweezers, to biological objects led to numerous Nobel Prizes [103]. Later, methods of statistical physics and physics-originated computational techniques, such as molecular dynamics, were also used to describe biological systems.

Physics can also help to reveal the operation principles by constructing simple models of complex structures. In the words of Phil Nelson (cited in [104]): “Physical models are often weirdly, unreasonably effective in stripping away the inessential from a biological system—and in displaying connections between things that seemed not obviously connected to our untrained imagination”. This is precisely what we have done in our works on mitochondrial proton-pumping complexes, which are overviewed in this paper. Our models elucidated the main physical mechanisms leading to biological functions. In other words, our works connected the physics of moving electrons and protons and their interactions to the biology of the mitochondrial respiratory chain.

This established connection can be explored in various medical applications. In this review, we addressed only two diseases associated with mitochondrial dysfunction: cancer and autism spectrum disorder. Specifically, we proposed that ROS overproduction in mitochondria can be caused by aberrations in the ECM and, in turn, serve as a trigger for somatic mutations in DNA. Correspondingly, monitoring the ECM status and mitochondrial function can lead to early cancer diagnostics. In particular, we have recently shown that the structural biomarkers of breast cancer can be found in the X-ray diffraction patterns of the tissues [105]. Our physical models can be extended to account for electron leakage in the proton-pumping complexes and corresponding ROS production.

We also suggested that the imbalance between classical and quantum brain functions in early childhood can lead to autism spectrum disorder. This imbalance can be caused by mitochondrial dysfunction, i.e., the disproportion of phosphates and pyrophosphates, which are the products of the ATP-ADP and ATP-cAMP cycles, respectively. Our models can be expanded to include both of these cycles. We can determine the production rates and their dependencies on external parameters and, therefore, recommend how the proper balance can be restored. It can lead to early diagnosis, treatment, and prevention of ASD if our hypothesis is proven true. We believe that even if it is not verified, research in these directions is worth pursuing, as it can lead to breakthroughs in various areas of human well-being.

## Data Availability

No new data were created or analyzed in this study. Data sharing is not applicable to this article.
